# Theta Activity Dynamics during Embedded Response Plan Processing in Tourette Syndrome

**DOI:** 10.3390/biomedicines11020393

**Published:** 2023-01-28

**Authors:** Paul Wendiggensen, Theresa Paulus, Annet Bluschke, Adam Takacs, Eszter Toth-Faber, Anne Weissbach, Tobias Bäumer, Christian Frings, Veit Roessner, Alexander Münchau, Christian Beste

**Affiliations:** 1 Cognitive Neurophysiology, Department of Child and Adolescent Psychiatry, Faculty of Medicine, TU Dresden, 01309 Dresden, Germany; 2 Institute of Systems Motor Science, University of Lübeck, 23562 Lübeck, Germany; 3 Doctoral School of Psychology, ELTE Eötvös Loránd University, 1053 Budapest, Hungary; 4 Institute of Psychology, ELTE Eötvös Loránd University, 1053 Budapest, Hungary; 5 Cognitive Psychology, Department of Psychology, University of Trier, 54286 Trier, Germany

**Keywords:** Tourette syndrome, motor processes, binding, theory of event coding, EEG

## Abstract

Gilles de la Tourette syndrome (GTS) is a neuropsychiatric disorder. Because motor signs are the defining feature of GTS, addressing the neurophysiology of motor processes is central to understanding GTS. The integration of voluntary motor processes is subject to so-called “binding problems”, i.e., how different aspects of an action are integrated. This was conceptualized in the theory of event coding, in which ‘action files’ accomplish the integration of motor features. We examined the functional neuroanatomical architecture of EEG theta band activity related to action file processing in GTS patients and healthy controls. Whereas, in keeping with previous data, behavioral performance during action file processing did not differ between GTS and controls, underlying patterns of neural activity were profoundly different. Superior parietal regions (BA7) were predominantly engaged in healthy controls, but superior frontal regions (BA9, BA10) in GTS indicated that the processing of different motor feature codes was central for action file processing in healthy controls, whereas episodic processing was more relevant in GTS. The data suggests a cascade of cognitive branching in fronto-polar areas followed by episodic processing in superior frontal regions in GTS. Patients with GTS accomplish the integration of motor plans via qualitatively different neurophysiological processes.

## 1. Introduction

Gilles de la Tourette syndrome (GTS) is a neurodevelopmental, multi-faceted neuropsychiatric disorder [1] with motor tics as its most characteristic sign [2]. Given the prominence of motor signs, GTS has often been viewed as a movement disorder. Recent years have witnessed a number of studies showing that this is unlikely to be the case [3]. Rather, it appears that the integration of perception and actions plays a critical role in the pathophysiology of GTS [4,5,6]. This notwithstanding, because tics as a motor sign are the defining feature of GTS, addressing motor processing in these patients, particularly regarding neurophysiological mechanisms, is a prerequisite for the understanding of this disorder.

As is the case for the integration of perception and action, the integration of motor subprocesses is also subject to so-called “binding problems” [7,8,9]. Such “binding problems” appear very relevant for the understanding of GTS, particularly with respect to perception–action integration [3]. In fact, within the Theory of Event Coding (TEC) [7], it has recently been demonstrated that perception–action binding is increased in GTS. Within the TEC, however, not only is perception–action integration detailed, but also how different features define a motor response are integrated to allow for the smooth unfolding of actions. In TEC, this is conceptualized as “action files” [10,11]. Action files can be examined in experimental procedures in which an action (A) is planned, but its execution has to be postponed until another action (B) is planned and performed. When there is strong binding of motor features constituting action A, it is demanding to execute another action B that shares some features with action A [10,11]. Very recent research in GTS, however, provided the first evidence that action file binding processes are not altered in adult GTS [12]. However, that study does not provide detailed insights into the functional neuroanatomical architecture associated with neural activity during action file coding and embedded action planning in patients with GTS and in healthy controls. This, however, is critical for various reasons:

In fact, it is likely that the functional neuroanatomical architecture associated with neurophysiological processes is different between patients with GTS and healthy controls because there are numerous reports that structural and functional brain organization is altered in these patients [13,14,15,16]. Particularly with respect to brain networks implicated in motor control, such alterations were reported [15,16]. Moreover, critically, the role of oscillatory brain activity and frequency band-specific processes as a major property of neural information processing [17,18,19,20] has not been evaluated in GTS. This is critical in the context of GTS because evidence is accumulating that this, especially theta band activity in fronto-striatal-thalamic loops, is relevant for the understanding of the pathophysiology of GTS [21,22,23,24,25,26,27,28,29,30]. Moreover, theta oscillations are important in action and motor control [31,32] because their biophysical properties are the basis for the integration of information across distant neural assemblies [17,31,33]. In the current study, we thus focused on theta frequency band activity (TBA) and examined the functional neuroanatomical architecture associated with TBA during action file coding in GTS. Based on the above considerations, we hypothesized that the functional neuroanatomical pattern of brain activation associated with action file binding effects in the theta band differs between patients with GTS and healthy controls. We assume that differences in the pattern of activities associated with action file binding effects in patients with GTS and controls are most likely evident in parietal and frontal regions because fMRI-based functional connectivity analyses revealed changes in GTS structures, particularly in fronto-parietal networks [34,35].

Importantly, considering the role of theta oscillations for the above-mentioned processes in GTS, we did not restrict our analyses to the period where action files are actually processed, but extended them to in-between trial/pre-trial periods in an action file coding experiment. The rationale is that previous data [36] suggest that TBA in particular in the pre-trial period is predictive for the strength/modulation of TBA within a trial in which motor response control processes are required. Such pre-trial TBA readings were interpreted to reflect some form of proactive control processes, i.e., processes needed to prepare the cognitive system for upcoming demands [37,38,39]. Theoretical approaches on prefrontal cortex functioning indicate that particularly fronto-polar regions play a role in “branching control” [40,41,42]. Such a branching of control enables the maintenance of a state/information that may be useful in the future to revert to a pending task following the completion of an ongoing one [41]. Such branching processes are assumed to precede processes of episodic control [41], comprising signals for guiding action selection. This episodic control is of central relevance for binding processes in the TEC-framework because these depend on the retrieval of information from episodic memory traces [43]. Based on previous findings of theta band activity in pre-trial intervals and above-mentioned theoretical approaches on prefrontal cortex functioning and information theory considerations [40,41,42], anterior orbito-frontal and anterior ventro-medial cortical areas are likely activated in the pre-trial interval. The fronto-polar region is closely connected to regions in the temporal cortex (temporal pole and superior temporal cortex) as well as more posterior regions in the ventro-lateral prefrontal cortex, including inferior frontal cortices [42,44,45]. Therefore, these regions may also be activated in the pre-trial phase. No clear hypotheses can be stated regarding the direction of pre-trial/within-trial correlations of TBA and whether there are differences between patients with GTS and healthy controls. However, given that structural neuroanatomical connections show widespread changes in patients with GTS [13,15] differences are likely. To calculate these correlations, we used the source-reconstructed activity as was done in previous work [36]. This is because the considerations outlined above suggest that the functional neuroanatomical structure is also of importance for a theoretically meaningful interpretation of possible correlations between pre-trial and within-trial theta band activity. Moreover, this approach confers the advantage that beamforming reduces residual variance in EEG data and, thus, increases the reliability of correlation analyses using neurophysiological data.

## 2. Materials and Methods

### 2.1. Patients and Controls

All patients and healthy controls provided written informed consent to participate in the study. The study was conducted in agreement with the Declaration of Helsinki (1964). From child or adolescent patients, a written informed consent of their legal guardians was obtained. The local ethics committee approved the study.

We investigated a group of *N* = 30 patients with GTS (*N* = 19 male patients, *N* = 11 female patients, mean age 20.67 ± 6.63 standard deviation (SD), range 12–35 years); part of these patients were also investigated in a previous study [12]. Adult patients (*N* = 18) were recruited from the specialized GTS outpatient clinic in the Department for Psychiatry and Psychotherapy of the University Medical Center Schleswig-Holstein, Campus Lübeck, Germany. Child and adolescent patients (*N* = 12) were recruited from the Department of Child and Adolescent Psychiatry at the University Hospital Dresden, Germany, and the Vadaskert Child and Adolescent Psychiatry Hospital and Outpatient Clinic in Budapest, Hungary. To assess the lifetime clinical information of each patient, we used a standardized clinical assessment, including a clinical neuropsychiatric interview, IQ testing and scoring of tic severity and obsessive-compulsive symptoms. To screen psychiatric co-morbidities, i.e., mood disorders and obsessive compulsive disorder (OCD), we used the Mini International Neuropsychiatric Interview (M.I.N.I.) [46] for participants over 17 years and the Mini International Neuropsychiatric Interview Kid (M.I.N.I. KID) [47] for participants from 9 to 17 years. The M.I.N.I. KID also evaluates the presence of attention deficit hyperactivity disorder (ADHD). The severity of ADHD of the adult patients was determined using the ADHD-Index and the DSM-ADHD-Scale of the German version of the Conners Adult ADHD Rating Scale [48]. Lifetime tics were evaluated by the Diagnostic Confidence Index [49]. Tic severity was identified using the Yale Global Tic Severity Scale (YGTSS) [50]. Premonitory urges were measured using the Premonitory Urge for Tic Scale (PUTS) [51]. We recorded a standardized video from each participant, which was independently scored by two experienced examiners using the Modified Rush Videotape Rating Scale [52] with a total tic score ranging from 0 to 20. When scores of the two examiners differed, a consensus was reached after discussing all relevant segments of the video. In addition to a Rush consensus score, we determined the tic frequency (tics/min). Depending on the participant’s age, OCD symptoms were evaluated using the Yale Brown Obsessive Compulsive Scale (YBOCS) [53] or the Children´s Yale Brown Obsessive Compulsive Scale (CY-BOCS) [54]. For IQ testing, we used either the short German version of the Wechsler Intelligence Scale for children (HAWIK-IV) for participants from 9 to 16 years [55] or the Wechsler Adult Intelligence Scale (WAIS) for participants over 16 years [56]. Handedness was determined using the Edinburgh Handedness Inventory [57].

We also investigated *N* = 30 healthy control participants (21 male patients, 9 female patients, mean age 20.93 ± 6.74 SD, range 12–35 years). Comparable to the patients, healthy controls also underwent clinical assessment that included a clinical neuropsychiatric interview, IQ testing, the M.I.N.I. or M.I.N.I. KID and the YBOCS or the CY-BOCS, and for adult participants, the ADHD-Index and the DSM-ADHD-Scale of the German version of the Conners Adult ADHD Rating Scale. Further, we also recorded a standardized video to determine total tic score and detect handedness. According to the interview assessment, six healthy controls had comorbidities. Three had depression in the past, one had panic attacks in the past and one had recurrent depression and panic attacks in the past. They had no clinically relevant psychiatric symptomatology during their study participation.

The clinical data of the Tourette patients are shown in Table 1. Eight GTS patients had depression in the past, and only one of them had a depressive episode at the time of testing. One had an anxiety disorder, and two had panic attacks in the past. Further, six GTS patients had a diagnosis of ADHD. One patient consumed cannabis on a regular basis. At the time of study participation, 7 of 30 patients were on medication, including aripiprazole (*N* = 5), tiapride (*N* = 1) and methylphenidate (*N* = 2). The mean IQ of the patient group was 102.9 (±13.4), and it was 110.9 (±9.22) for the control group (t(51) = −2.69, *p* < 0.01). Two patients and three healthy control participants were left-handed.

### 2.2. Task

**Figure 1:**
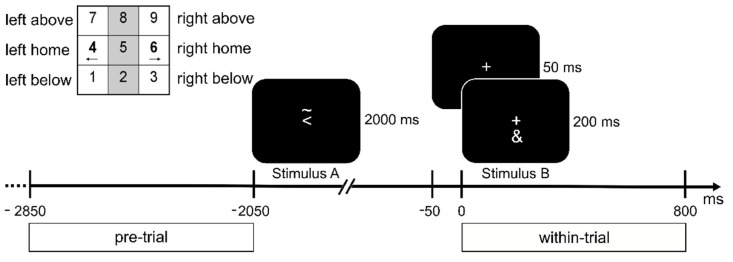
Illustration of the task. Presented stimuli are visualized on a time scale relative to Stimulus B. A fixation cross was shown at all times. In the top left corner, the layout of the response keys (num pad on a keyboard) is shown. Participants had to respond to the Stimuli with the corresponding left or right index fingers. The figure depicts a feature overlap trial: both Stimulus A and B require a response with the left hand, as indicated by the arrowhead in Stimulus A and the ‘&’ symbol in Stimulus B (refer to Methods). Below the time axis, the time frames chosen for the pre- and within-trial interval are outlined.

The “R-R task” paradigm [11,58] was previously used to investigate action file binding processes [59]. The experiment was programmed in Presentation (Neurobehavioral Systems Inc., Berkeley, CA, USA) and is visualized in Figure 1.

Stimuli were shown in white on a black background on a 17-inch CRT screen at a viewing distance of 60 cm from the participants. Each trial consisted of two tasks nested within each other (stimulus–response pattern ABBA). A fixation cross was displayed for 50 ms at the beginning of each trial. Then, Stimulus A was presented for 2000 ms. The response for Stimulus A (S1) had to be planned and withheld until the response for Stimulus B (S2). After the presentation of Stimulus A, a fixation cross was displayed for 50 ms and followed by Stimulus B, which was presented for 200 ms. Stimulus A consisted of a left-pointing or right-pointing arrowhead and the symbol “~” either above or below the arrowhead. The arrowhead indicated the response hand (i.e., left or right hand), whereas the symbol “~” indicated the response direction (i.e., up or down). A series of three consecutive button presses was required for Stimulus A: first, the home key on the side described by the arrowhead (i.e., left or right), followed by a press on the key above or below in the direction of the asterisk, and lastly, the home key had to be pressed for a second time (see Figure 1). Stimulus B was either the symbol “&” or “#”, which indicated a left or right button press, respectively. Stimulus B required a single button press on the response side corresponding to the stimulus. The numerical pad on the keyboard was used for the participant’s responses. The buttons 1, 4 and 7 (below, home and above) were used for the left response direction, and the buttons 3, 6 and 9 were used for the right response direction. The participants were instructed to leave their index fingers on the corresponding sides of the numerical pad. If Stimulus A consisted of a right-pointing arrowhead with the symbol “~” below it and Stimulus B was the symbol “&”, the correct response sequence was 4 (left home key)—6 (right home key)—3 (below)—6 (right home key). Participants performed a practice round of 40 trials before a total of 256 trials were presented in four blocks. Of these 256 trials, half of the trials revealed a feature overlap in the response side (same hand used for the response for Stimulus A and B), and the other half of the trials revealed no feature overlap (different hands used for Stimulus A and B).

### 2.3. EEG Recording and Analysis

EEG data were recorded using 60 Ag/AgCl electrodes with an equidistant cap layout (EasyCap, Wörthsee, Germany) at a sampling rate of 500 Hz. A “QuickAmp” amplifier and the “Brain Vision Recorder” software (Brain Products, Gilching, Germany) were used for the recording. The ground and reference electrodes were placed at the coordinates of θ = 58, φ = 78 and θ = 90, φ = 90, respectively. Electrode impedances were kept below 5 kΩ. The EEG data were pre-processed using the Brain Vision Analyzer (Brain Products, Gilching, Germany). The data were down-sampled to 256 Hz and filtered between 0.5 Hz and 40 Hz at an order of 8. Additionally, a 50 Hz notch filter was applied. Consecutively, the data were re-referenced to the average activity of all electrodes. EEG data were manually inspected, and technical artefacts were removed. Eye blinks, lateral eye movements and periodical artefacts (i.e., cardiovascular activity) were removed using an independent component analysis (ICA, Infomax algorithm) after visual inspection of the component topographies and time-courses. The cleaned data sets were transferred to the Fieldtrip Toolbox [60] running in Matlab R2019a (Mathworks, Inc., Massachusetts, United States). The data were segmented based on the presentation of Stimulus B (−5 to 3 s) for both overlap levels (feature overlap vs. no-feature overlap). For further analysis, a pre-trial interval (−2850 to −2050 ms) prior to the presentation of Stimulus A and a within-trial (0 to 800 ms) after the presentation of Stimulus B were chosen. The duration of these intervals was set to 800 ms, as this period allowed for at least four theta cycles at a central frequency of 5.5 Hz (see Beamforming section). Automated artefact rejection was applied to all segments with amplitudes higher than 100 μV and lower than −100 μV. Furthermore, segments with activity below 0.5 μV over a time period of 100 ms were removed from the data.

For the within-trial interval, segments were divided into a feature overlap and no-feature overlap condition. For the pre-trial interval, both the feature overlap and no-feature overlap trials were selected, as the participant could not be aware of the trial type before the presentation of the stimuli. For the pre- and within-trial periods, time-frequency (TF) analyses on the segments were performed using Morlet wavelets (𝑤) as presented below:

(1) $$w\left(t,f\right)=A\exp\left(-\frac{t}{2\delta_{t}^{2}}\right)\exp\left(2i\pi ft\right),$$

with t = time, $A={\left(\delta_{t}\sqrt{\pi }\right)}^{-1/2}$, $\delta_{t}$ = wavelet duration, and $i=\sqrt{-1}$. A ratio of between central frequency ($f_{0})$ and the width of the Gaussian shape ($\delta_{f}$)=5 was used. The average power over the theta frequency band was calculated for each electrode and time point. To examine the binding effect, the TBA in the within-trial period was compared between the feature overlap and no-feature overlap trials. *T*-tests between the feature overlap and no-feature overlap conditions were computed for every time point between 0 and 800 ms relative to Stimulus B onset separately for controls and GTS patients.

### 2.4. Beamforming Analysis

Source-level activity was reconstructed from the sensor-level EEG data using a multi-step beamforming approach as applied in previous studies [36]. In the first step, a Dynamic Imaging of Coherent Sources (DICS) [61] beamformer was applied. For this, a Fast Fourier Transform (FFT) was calculated at the central frequency of 5.5 Hz with a taper of 1.5 Hz. From the cross-frequency spectrum, common spatial filters were computed for the feature overlap condition and the no-feature overlap conditions for both the control and the patient group. The data were projected into the source space onto an equally spaced 1 cm grid created from an MNI (Montreal Neurological Institute) space. The projection of the data was based on the forward model included in the FieldTrip toolbox. For the control and the patient group, theta power values were extracted for the pre-trial and the within-trial interval. The Neural Activity Index (NAI) was calculated for the pre-trial period by dividing the source values by the local noise estimates [62]. For the within-trial interval, a contrast between the feature overlap and the no-feature overlap condition was calculated and normalized on the total power of both conditions.

After DICS-beamforming, the clusters of theta activity in the reconstructed source data were determined by applying a Density-Based Spatial Clustering of Applications with Noise (DBSCAN) [63] in Matlab. Using the DBSCAN algorithm, further analyses could be restricted to voxels within functional neuroanatomical regions with high theta activity. For this, power values were thresholded at the top 1% of the power distribution within regions labeled in the Automatic Anatomical Labelling (AAL) [64] atlas. For the DBSCAN, the minimum cluster size was set to seven voxels, and the epsilon value was set to twice the edge length of each voxel in order to detect neighboring voxels. The clusters were visually inspected and selected for further analysis based on anatomical label and cluster size (voxels included in the cluster). Subsequent beamforming analysis was restricted on the selected voxels.

In the last step, a Linear Constraint Minimum Variance (LCMV) [62] beamformer was applied to retrieve the TBA time course in the selected voxels. The pre-processed data were multiplied with the spatial filter computed on the covariance data of the averaged data for both the control and the patient group. Time-frequency analyses were calculated using Morlet wavelets with the same parameters as before (see Section 2.3). Power values were averaged over all voxels included in each cluster, resulting in a single time-frequency course for each TBA cluster. As a measure of the binding effect, the difference between the feature overlap and no-feature overlap condition was calculated. Pearson correlations were calculated for each time point between clusters of the pre-trial and within-trial periods. The resulting correlation matrices visualize the time course of the two clusters on the axes, and thus, each value in the matrix represents the correlation between the respective time points in the two clusters. To reduce the likelihood of false-positive findings due to the large number of calculated correlations, the Benjamini-Hochberg [65] method was applied to control the false discovery rate (FDR). In addition to the FDR correction, only q-values (*p*-values corrected for FDR) smaller than 0.01 were regarded as significant.

### 2.5. Statistical Analysis

The statistical analyses on the behavioral data were conducted using R [66]. The mean accuracy (percentage of correct responses) and mean reaction times (RTs) for correct responses were calculated for Stimulus B (immediate response). For Stimulus A (planned response), mean RTs and mean accuracy were calculated over all three required responses cumulatively. Mixed effects ANOVAs were calculated for RT and accuracy on both stimuli with the within-factor task (feature overlap vs. no-feature overlap) and between factor groups (GTS vs. HC). Bayesian linear models (BF10) analogous to the mixed effects ANOVAs were calculated with the “BayesFactor” package [67]. The Bayes Factor (BF) provides the marginal likelihood of the model compared to the null model. Below a Bayes Factor of 1, both models are equally likely, whereas a Bayes Factor < 1 favors the null hypothesis, and a Bayes Factor > 1 favors the alternative hypothesis [68]. A Bayes Factor < 1/3 or > 3 provides moderate evidence for H0 and H1, respectively, whereas a Bayes Factor of 10 can be interpreted as strong evidence for H1 (analogously, a BF of 1/10 provides strong evidence for H0).

## 3. Results

### 3.1. Behavioral Data

An ANOVA including the within-factor task (feature overlap vs. no-feature overlap) and between-factor group (GTS vs. HC) on the reaction time to Stimulus B revealed the significant main effect of task condition (F_1,58_ = 9.38, *p* = 0.003, *η^2^_g_* < 0.01). A BF_10_ = 10.28 ± 1.3% can be interpreted as strong evidence for this model. RTs were higher in the feature overlap (*M* = 575 ms ± SE = 25) compared to the no-feature overlap condition (*M* = 558 ms ± SE = 26), indicating a binding effect for the response times. There was no significant main effect of group (F_1,58_ = 0.57, *p* = 0.45, *η^2^_g_* = 0.01, BF_10_ = 0.78 ± 0.5%) and no significant interaction effect of task and group (F_1,58_ = 0.34, *p* = 0.56, *η^2^_g_* < 0.001, BF_10_ = 0.29 ± 0.8%). An ANOVA on the accuracy in response B with the within-factor task and the between-factor group showed no significant main effects for group (F_1,58_ = 1.18, *p* = 0.28, *η^2^_g_* = 0.01, BF_10_ = 0.44 ± 0.8%) or task (F_1,58_ = 0.11, *p* = 0.74, *η^2^_g_* < 0.001, BF_10_ = 0.20 ± 1.1%). There was no significant interaction effect of group and task (F_1,58_ = 0.20, *p* = 0.66, *η^2^_g_* = 0.001, BF_10_ = 0.28 ± 1.5%). An ANOVA on the cumulated response times for Stimulus A with the within-factor task and between-factor group did not reveal significant main effects of group (F_1,58_ = 1.13, *p* = 0.29, *η^2^_g_* = 0.02, BF_10_ = 0.77 ± 3.1%) and task (F_1,58_ = 1.54, *p* = 0.22, *η^2^_g_* < 0.001, BF_10_ = 0.38 ± 0.8%). Additionally, there was no significant interaction effect of group and task (F_1,58_ < 0.01, *p* = 0.95, *η^2^_g_* < 0.001, BF_10_ = 0.25 ± 0.8%). In the ANOVA on the overall accuracy for Stimulus A, there were no significant main effects of group (F_1,58_ = 0.64, *p* = 0.43, *η^2^_g_* = 0.01, BF_10_ = 0.64 ± 2.7%) and task (F_1,58_ = 2.03, *p* = 0.16, *η^2^_g_* < 0.01, BF_10_ = 0.47 ± 1.2%). There was no significant interaction effect of group and task (F_1,58_ = 0.13, *p* = 0.71, *η^2^_g_* < 0.001, BF_10_ = 0.27 ± 1.6%).

### 3.2. Neurophysiology

**Figure 2:**
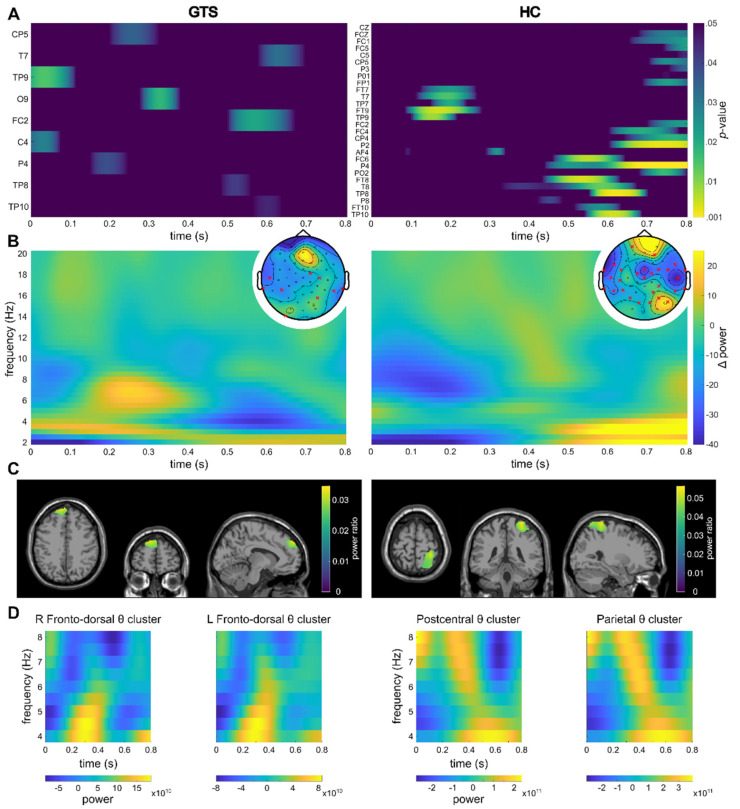
Within-trial results. For all subplots, the left side of the figure refers to the GTS group, whereas the right side refers to the HC group. (**A**) Significant channels and timeframes for channel-wise *t*-tests on theta power between the feature overlap and no-feature overlap conditions are shown. Warmer colors indicate smaller *p*-values (colormap threshold at *p* < 0.05). (**B**) Sensor-level time frequency representation of the difference between the feature overlap and no-feature overlap conditions over the channels showing significant differences in the theta band are shown (please refer to (**A**)). Topoplots show the mean theta power over the entire segment as well as the significant channels (indicated by a red cross). (**C**) Clusters as identified by the DBSCAN algorithm are shown. The identified clusters (refer to Section 3) are visualized on a template MRI. For voxels within the cluster, the source ratio (difference between the feature overlap levels divided by the sum of the overlap levels) is visualized. (**D**) Time-frequency representations of the theta band in in the respective clusters.

For the within-trial interval, significant differences in TBA between the feature overlap and no-feature overlap conditions were found for multiple electrodes for both the HC and GTS group (Figure 2A). The time-frequency representations over the electrodes showing significant differences can be found in Figure 2B. For the source-level data and the within-trial interval, theta activity clusters in the right inferior postcentral cortex and the right superior parietal cortex were found in the HC group (Figure 2C). In the GTS group, a cluster in the left fronto-dorsal cortex was established as well as a smaller cluster in the right fronto-dorsal cortex.

**Figure 3:**
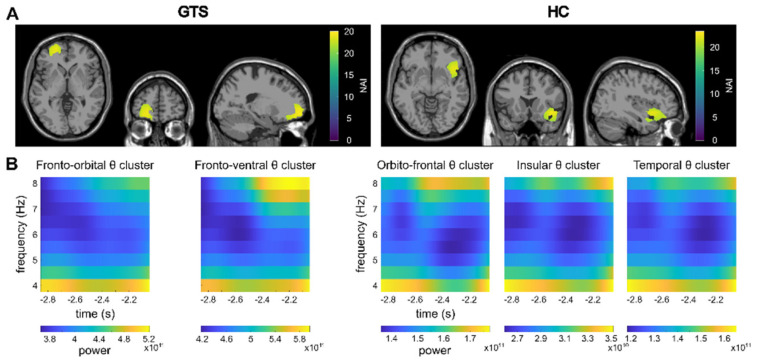
Pre-trial results. (**A**) Clusters as identified by the DBSCAN algorithm in the pre-trial period are shown. The identified clusters (refer to *3. Results*) are visualized on a template MRI. Please note that the clusters are adjacent to each other and therefore cannot be distinguished in this visualization. The Neural Activity Index (NAI) is given for voxels within the clusters with warmer colors representing higher values. (**B**) Time-frequency representations of the theta band in the identified clusters are shown.

For the pre-trial interval, the TBA cannot be compared to another condition or interval, as the pre-trial interval encompasses the period that is typically used as a baseline. Hence, it is not possible to establish whether the TBA activity in the pre-trial interval differs from random signals on the electrode level. As the clusters identified on the source-level show significant correlations even after FDR-correction was applied, we conclude that the activation in the identified regions is meaningful, as the correlations between random/non-meaningful signals multiplied with a filter created from the signals (see Section 2.3—LCMV beamforming) cannot be significant. In the GTS group, clusters of theta activity were revealed within the left medial frontal cortex and the left fronto-ventral cortex in the pre-trial interval (Figure 3).

**Figure 4:**
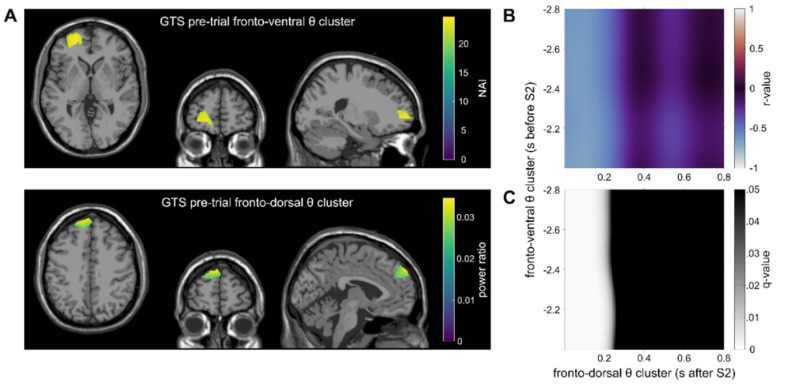
Main results of the correlations in the GTS group are shown. (**A**) Theta clusters: the top figure depicts theta band activity in the fronto-ventral cluster as identified by the DBSCAN algorithm in the pre-trial interval. The Neural Activity Index (NAI) is shown for voxels within the cluster. The bottom figure depicts the within-trial left fronto-dorsal theta cluster. For voxels within the cluster, the source ratio (difference between the feature overlap levels divided by the sum of the overlap levels) is color-coded. (**B**) The correlation map between the clusters from Figure 3A is shown. The correlation map shows the Pearson correlation for each time point between the pre- and within-trial tasks (color represents the *r*-value). (**C**) FDR-corrected *q*-values for the correlation map Figure 3B. Black areas indicate no significant correlation (*q* > 0.05), whereas bright areas indicate a significant correlation.

The correlation map between the pre-trial ventral frontal theta cluster and the within-trial fronto-dorsal theta cluster revealed a significant negative correlation in the GTS group (Figure 4; please note that only significant correlation maps are shown). In the correlational analyses, strong correlations between the fronto-ventral theta activity at all time points during the pre-trial and the theta activity in the fronto-dorsal cluster in the time frame from 0 to approximately 200 ms after the presentation of Stimulus B were evident (*r_max_* = −0.53; *r_min_* = −0.67 in *q* < 0.01). This indicates that higher pre-trial TBA in the ventral frontal cortex is associated with lower TBA in the fronto-dorsal cortex during the presentation of Stimulus B.

For the control group, the DBSCAN analysis of the beamformed data (contrast feature overlap vs. no-feature overlap) revealed clusters of TBA in the orbitofrontal cortex, the insula and the temporal cortex in the pre-trial interval (Figure 3). However, no significant correlations between the pre-trial and within-trial clusters were found.

## 4. Discussion

In the current study, we examined the functional neuroanatomical architecture of theta band activity related to action file processing in patients with GTS and healthy controls. Other than taking more conventional approaches to EEG data analysis, we did not restrict the data analysis to the period, in which action files are processed, but extended the analysis to the pre-trial period.

In keeping with a previous study [12], behavioral data (reaction times and accuracy) did not show differences in action file binding effects between patients with GTS and controls. However, neurophysiological dynamics in GTS and healthy controls revealed qualitative differences in brain organization and/or differences in the strategies applied rather than differences in the efficacy of action file processing. The data show that in the within-trial phase, action file binding effects in TBA were associated with superior parietal regions (BA7) and the precuneus (BA7) in the control group. In the GTS group, superior frontal regions (medial and lateral) (BA9, BA10) were found. This group-specific pattern of brain regions associated with action file binding effects, together with a lack of group differences in the behavioral data, suggests that patients with GTS and healthy controls accomplish action file binding via qualitatively distinct neural processes using different strategies.

Thus, in healthy controls, the superior parietal cortex (BA7) was associated with action file binding effects in the theta band. BA7 was shown to be important for visuo-motor coordination [69,70,71,72,73,74], especially in bimanual tasks [75], which is also the case in the applied action file binding experiment. In addition, BA7 was found to comprise separate, interconnected functional systems representing motor features [69,71,73]. The used action file experiment incorporates an ABBA design for response execution, in which an action (A) is planned, but its execution has to be postponed until another action (B) is planned and performed. Feature overlap of these actions A and B varies [10,11]. Because the inter-independency of motor features plays a major role in action file binding effects, the obtained association of the action file binding effect in the theta band with BA7 likely comprising interconnected functional systems representing motor features is plausible [69,71,73]. The data may therefore be interpreted such that in healthy controls, the representation of motor features is critical during action file binding.

In contrast to the healthy control group, action file binding effects in the theta band were associated with lateral regions of the superior frontal gyrus (BA9, BA10) in GTS patients. To interpret this finding, overarching theoretical conceptions on the functional architecture of the prefrontal cortex are helpful, particularly information on theoretical approaches to prefrontal cortex functioning [41,42]. According to this, there are three nested processing levels: (i). branching, (ii). episodic and (iii). contextual processes, which are implemented from anterior (polar) regions to more posterior regions of the lateral prefrontal cortex [41]. The anterior lateral prefrontal cortex is especially involved in episodic control, which is defined as control of ongoing behavior that is composed of multiple successive temporal frames or episodes [41]. Binding in the TEC framework strongly relies upon episodic retrieval [43]. This is related to the structure of experimental approaches assessing binding effects, in which each trial consists of a series of events, where stimuli are presented and/or responses have to be executed. Therefore, single trials in typical implementations of experiments examining binding processes build their own ‘episodic history’. This is also the case in the applied action file paradigm, where each trial has an ABBA design for different to-be executed responses [10,11]. Apparently, episodic processing of the ABBA response history is central in patients with GTS during action file coding. In light of the findings obtained for the healthy control group discussed above, the entire data pattern may be interpreted such that healthy controls and patients with GTS use different strategies to accomplish action file tasks. For healthy controls, the processing of different motor feature codes is central for action file processing; for patients with GTS, it is episodic processing.

Another important focus of the current study was on the analysis of theta activity in the pre-trial period and its relation to theta activity associated with action file binding effects. The pre-trial period likely reflects proactive control processes [36] similar to cognitive branching processes [41]. Because cognitive branching precedes processes of episodic control [41] playing a role in binding processes [43], the inter-relation of pre-trial and within-trial activity is of particular interest. In addition, the above discussion based on theoretical conceptions of the functional relevance of prefrontal structures, which suggests that episodic processing is most relevant during action file coding in GTS, underlines the relevance of this analysis. Considering the functional neuroanatomical level, correlations between pre-trial and within-trial activity were thus calculated using source-reconstructed TBA [36].

In patients with GTS, left-sided fronto-polar areas of the superior and middle frontal gyrus (BA10, BA11) were activated in the pre-trial interval. Fronto-polar region were implicated in cognitive branching processes [41,42] considered to enable/maintain a state/information that may be useful in the future [41]. This cognitive branching precedes episodic control processes associated with anterior regions of the lateral prefrontal cortex found to be associated with theta band activity during action file coding (i.e., within a trial) [41]. Importantly, the correlation analysis shows that theta-related pre-trial activity in the fronto-polar region was significantly negatively correlated with theta activity reflecting action file binding in GTS. As shown in Figure 4, theta activity in the entire analyzed period in the pre-trial interval was correlated with theta band activity in the first 200 ms after time point zero (i.e., within-trial activity after the presentation of stimulus B). Thus, stronger theta band activity during cognitive branching is related to lower episodic processing during action file coding in these patients. However, given the direction of the correlation with TBA during action file coding, it is possible that this activity reflects some form of preparatory processes or pre-activation of memory representations of motor response features. If these response features are pre-activated, they are more readily usable for the cascaded execution of responses (cf. ABBA trial design). This interpretation is in keeping with the notion that pre-trial TBA activity can increase the efficiency of motor response control processes [36]. Apparently, this interpretation has conceptual similarities with cognitive branching, which was proposed to be a function of the fronto-polar cortex [41]. The correlation therefore suggests that cognitive branching processes are directly relevant, i.e., may inform episodic processing of ABBA response history in patients with GTS. This contrasts with the healthy controls, where no significant correlations between pre-trial and within-trial TBA were obtained. This dissociation can only tentatively be interpreted. However, it seems that healthy controls, unlike patients with GTS, do not need to employ proactive control or cognitive branching process to still yield similarly high behavioral performance than patients with GTS. This underlines the above interpretation that patients with GTS accomplish action file coding and the integration of motor features to coherent action via qualitatively different neurophysiological processes.

In summary, in keeping with previous data, whereas behavioral performance during action file processing did not differ between GTS and controls, the underlying patterns of neural activity were profoundly different. Regarding within-trial processing, superior parietal regions (BA7) were predominantly engaged in healthy controls, but superior frontal regions (BA9, BA10) in GTS indicate that processing different motor feature codes is central for action file processing in healthy controls, whereas episodic processing is more relevant in GTS. Relations of pre-trial/within trial activity suggested a cascade of cognitive branching in fronto-polar areas followed by episodic processing in superior frontal regions in GTS. The findings show that future studies in patients with GTS should not only consider quantitative differences, but also should put emphasis on qualitatively different patterns of neural activity associated with GTS. Through the combined analysis of qualitative and quantitative differences, a more holistic picture of pathophysiological changes will be revealed, ultimately leading to a better conception of GTS and its treatment.

## Figures and Tables

**Table 1:** Clinical characteristics of the patients with GTS included in the study.

Patient	Age	Sex	IQ	Disease, Duration, Years	DCI (0–100)	YGTSS Total (0–100)	YGTSS Tics (0–50)	Motor Tics/Min	Rush Score (0–20)	PUTS (9–36)	YBOCS or CY-BOCS (0–40)	Medication
1	35	Female	110	28	44	17	7	3.9	3	15	10	Aripiprazol
2	28	Female	98	23	95	51	31	39	13	32	15	Aripiprazol, methylphenidat, amitriptyline
3	18	Male	117	8	43	17	7	4.5	10	13	0	
4	20	Male	117	15	56	58	28	17.3	13	27	0	Aripiprazol
5	28	Male	79	20	72	61	21	24.8	9	28	14	Aripiprazol
6	24	Male	112	9	63	67	27	8.7	11	22	9	
7	18	Male	85	11	55	50	30	42.2	10	17	0	
8	29	Male	106	15	79	n.a.	n.a.	29.4	13	24	13	
9	22	Female	80	18	100	46	26	30.7	13	18	0	
10	35	Female	96	22	76	47	27	40	14	22	0	Tiapride
11	26	Female	97	14	39	55	15	19.8	12	19	0	
12	23	Female	84	16	50	37	7	41.2	12	18	0	
13	22	Female	96	17	46	20	10	10.5	4	24	0	
14	26	Female	115	19	56	25	25	56.4	9	16	5	
15	23	Male	111	16	64	31	21	62.5	14	27	0	
16	30	Male	112	23	54	54	24	85.2	14	13	0	
17	19	Female	117	11	35	30	10	22.3	11	13	0	
18	20	Male	100	7	59	58	28	68	16	28	0	Aripiprazol
19	16	Male	86	-	-	45	25	2	3	16	7	
20	12	Male	101	-	-	32	22	7.6	7	16	-	
21	12	Male	105	-	-	28	18	2.4	3	11	-	
22	18	Male	98	7	40	13	13	21.4	10.5	-	14	Methylphenidate
23	16	Male	123	11	-	39	19	77.2	13.5	24	2	
24	17	Male	128	-	43	9	9	49.2	9.5	20	2	
25	16	Male	112	4	25	4	4	10.4	4	14	7	
26	13	Female	110	8	34	31	11	38.2	10.5	0	0	
27	14	Female	105	5	36	5	5	31.8	7.5	0	0	
28	14	Male	76	5	31	8	8	19.4	8	0	0	
29	13	Male	108	8	54	47	27	101.6	17	19	0	
30	13	Male	102	-	42	7	7	13.4	3.5	19	0	
MEAN	20.7	-	102.9		53.5	34.2	17.7	32.7	9.9	17.8	3.5	

DCI = Diagnostic Confidence Index; PUTS = Premonitory Urge for Tics Scale; YBOCS = Yale Brown Obsessive Compulsive Scale; CY-BOCS = Children’s Yale-Brown Obsessive-Compulsive Scale; YGTSS = Yale Global Tic Severity Scale.

## Data Availability

The data that support the findings of this study are available from the corresponding author upon reasonable request.
